# A review of acoustofluidic separation of bioparticles

**DOI:** 10.1007/s12551-023-01112-2

**Published:** 2023-08-29

**Authors:** Fria Hossein, Panagiota Angeli

**Affiliations:** Department of Chemical Engineering, University College London, Torrington Place, WC1E 7JE, London, UK

**Keywords:** Acoustofluidics, Microfluidics, Separations, Particles, Cells

## Abstract

Acoustofluidics is an emerging interdisciplinary research field that involves the integration of acoustics and microfluidics to address challenges in various scientific areas. This technology has proven to be a powerful tool for separating biological targets from complex fluids due to its label-free, biocompatible, and contact-free nature. Considering a careful designing process and tuning the acoustic field particles can be separated with high yield. Recently the advancement of acoustofluidics led to the development of point-of-care devices for separations of micro particles which address many of the limitations of conventional separation tools. This review article discusses the working principles and different approaches of acoustofluidic separation and provides a synopsis of its traditional and emerging applications, including the theory and mechanism of acoustofluidic separation, blood component separation, cell washing, fluorescence-activated cell sorting, circulating tumor cell isolation, and exosome isolation. The technology offers great potential for solving clinical problems and advancing scientific research.

## Introduction

The separation of bioparticles in mixtures has several important applications in various fields, such as biotechnology and pharmaceuticals, including purification of biological molecules, cell separation and analysis, environmental analysis, food and agriculture, as well as diagnostic and clinical applications (Yohannes et al. 2011, Vitorino et al. 2021). The separation of bioparticles in a mixture can be achieved through various methods such as centrifugation (Li et al. 2014), chromatography (Persson et al. 2004), filtration (Jubery et al. 2014; Hossein 2020; Salipante 2023), and flocculation (Van Hee et al. 2006). Centrifugation is a technique in which bioparticles of different sizes and densities can be separated by spinning them at high speeds. The centrifuge applies a powerful force to the mixture, which causes denser (heavier) bioparticles to separate and sediment at the bottom, while lighter particles remain in the supernatant. Chromatography is a separation technique in which bioparticles can be separated based on their physical and chemical properties, such as size, charge, and affinity, by passing their mixture through a stational phase. Filtration utilizes membranes with suitable pore sizes to retain the larger molecules while allowing smaller ones to pass through. These techniques can be used to obtain high-purity fractions of bioparticles. Recently, there has been a focus on the isolation of cells and bioparticles for various biological and biomedical purposes by using acoustic techniques. Acoustics can advance production in biotechnology by enhancing separation of cells and particles in complex mixtures (Martens and Demain 2017; Ventola 2015; Ramos et al. 2017). Wu et al. (2019) discuss the principles and various approaches of acoustofluidic separation, along with their traditional and emerging applications, including blood component separation, and cell washing. The authors emphasize the potential of acoustofluidics for solving clinical problems and advancing scientific research as a solution to clinical problems. Acoustofluidic systems have been designed to isolate particles with different sizes (Barani et al. 2016; Connacher et al. 2018; Wang 2021; Hall 2020) as well as particles with various mechanical properties (Friend and Yeo 2011; Laurell et al. 2007). Acoustic techniques could damage the cells or particles if the frequency of propagated waves is not carefully selected. This can be avoided by selecting frequencies in a similar ranges to those used in ultrasound imaging (Hossein 2019). Combining acoustic techniques with microfluidics, researchers have created compact systems that can effectively isolate, concentrate, and filter bioparticles (Haber and Velculescu 2014; Sajeesh and Sen 2014; Tauro et al. 2012). These systems have numerous benefits such as increased precision in separation, low power and reagent usage, small size separations, and minimal sample volume requirements, reduced costs, as well as the potential for disposability. Consequently, they have the potential to serve as point-of-care diagnostic tools (Wei et al. 2023; Rufo et al. 2022). Common separation methods can typically be categorized into either labeled or label-free techniques, as well as active or passive methods depending on the separation mechanism employed (Surendran et al. 2021; Alnaimat et al. 2018). Passive methods for separation include filtration, pinch flow margination, deterministic lateral displacement, and surface affinity-based separation (Gossett et al. 2010). Usually, passive methods involve more straightforward equipment setups, but active methods offer greater flexibility and can achieve superior separation resolution by leveraging differences in mechanical, electric, magnetic, and acoustic properties (Al-Ali et al. 2022). The integration of microfluidics and acoustofluidics allows for the precise sorting and separation of different types of bio-particles, such as cells, bacteria, or viruses. By applying acoustic forces to the fluid sample within the microchannel, these particles can be directed to different regions depending on their size, shape, or other properties. One key advantage of this combination is the ability to achieve high-throughput separation in a rapid and efficient manner. A comparison of available microfluidic methods for separating exosomes from blood or other biological fluids is presented in Table 1.

**Table 1:** A comparison of separation performance among various exosome separation methods is presented

Methods	Isolation principle	Yield (%)	Purity (%)	Pros	Cons	Reference
Ultracentrifugation	Density, size	5–50	23–70	Eligible for processing large volume samples, unbiased isolation	Exosome fusion, soluble protein contamination	Tauro et al. (2012), Helwa et al. (2017)
Density gradient centrifugation	Density differences	25–50	Not described	Lower levels of contamination from soluble proteins, unbiased isolation	Additional buffer preparation required	Chavez and Summers (2012), Greening et al. (2015)
Ultrafiltration	Size difference	14–35	70–82	Unbiased isolation	Low soluble protein removal rate, exosomal structure damage, protein aggregate	Muller et al. (2014), Lobb et al. (2015)
Immuno-magnetic isolation	Antibody capture and magnetic force	13–60	26–78	Low soluble protein contamination, eligible for specific exosome subpopulation isolation	Limited availability of robust capture antibodies, additional washing and preparation steps needed, may lose the full functionality of exosomes after elusion	Balaj et al. (2015), Qi et al. (2016)
Exo-Quick	Precipitation	40–80	28–87	Unbiased isolation, low structural damage	Contamination from soluble proteins	Taylor and Yang (2011), Tang et al. (2017)
Field flow fractionation	Size difference	Not described	Not described	Ability to isolate. Subsets of exosomes	Small volume samples (100 μL), lengthy procedure	Zhang et al. (2018)
Microfluidic methods Microfluidic immunoaffinity	Antibody capture	42–94	87–97	Low soluble protein contamination, eligible for specific exosome subpopulation isolation	Limited availability of robust capture antibodies, additional washing and preparation steps needed	Chen et al. (2010), Kanwar et al. (2014)
Acoustofluidics	Size and acoustic contrast factor	82	98	High exosome integrity, unbiased isolation, no requirement of additional reagent and washing steps	Soluble protein contamination	Wu et al. (2017)

Overall, Table 1 is offering insight into the yield, purity, biocompatibility, and throughput (flow rate) of different approaches and how they compare in a given application (Wu et al. 2017). Acoustofluidic separation has found extensive use in many applications, ranging from the isolation of rare circulating biomarkers to the differential focusing and separation of nanoparticles. The applications of acoustofluidic separations includes:

(1) Medical diagnosis and research: Acoustofluidic separations can be used for the separation of cells and biomolecules of different sizes and densities, which is useful for medical diagnosis and research (Li and Huang 2018). (2) Drug discovery: Acoustofluidic separations can be used for high-throughput drug discovery by separating target cells or molecules from a complex mixture (Nasiri et al. 2020). (3) Environmental monitoring: Acoustofluidic separations can be used for the separation of microorganisms and pollutants in environmental monitoring (Akiyama et al. 2020). (4) Food processing: Acoustofluidic separations can be used for the separation and purification of food ingredients, such as proteins and enzymes (Xie et al. 2020). (5) Industrial production: Acoustofluidic separations can be used for the separation and purification of various industrial materials, including chemicals, metals, and nanoparticles (Xie et al. 2020). (6) Water treatment: Acoustofluidic separations can be used for the removal of particles and impurities from water, such as bacteria, viruses, and microplastics (Chen et al. 2020). Acoustofluidic separation can effectively handle bioparticles of varying sizes, ranging from tens of nanometers to several hundred micrometers, which is significant for applications (Hao et al. 2020). Many biological targets, including those targeted for liquid biopsy development, fall within this size ranges. Liquid biopsies are non-invasive blood tests that are an alternative to traditional tissue biopsies (Hao et al. 2020; Yang et al. 2018). Liquid biopsies can not only diagnose diseases at an early stage but also identify specific genetic mutations, enabling doctors to tailor treatments and monitor patients’ responses (Siravegna et al. 2017).

Acoustofluidic separation techniques, have high precision and versatility, and have the potential to expand the separation capabilities in traditional applications (Xie et al. 2019b). 

This review aims to introduce acoustofluidic separation to a wider audience and presents the underlying theory, and comparisons with different technologies, and discusses current and future applications of acoustofluidic separation. Rather than encompassing a wide range of applications in the biomedical and bioanalytical fields, this review focuses on the acoustofluidic separation of cells and bio nanoparticles. It begins with an introduction to the basics of the theory and mechanism of acoustofluidic separation, before shedding light on the recent advancements of the technology in biological applications, and finally, discussing the challenges and prospects of this field. Table 2 presents the introduction of the physical quantities and experimental parameters used in this review.

**Table 2:** Physical quantities and experimental parameters

Parameters	Descriptions	Units
*AC*	Alternative current	Volt
*IDTs*	Interdigital transducer	mm
*f*	Frequency	Hz
*C*	Speed of sound	m/s
*C*_1_	Acoustic wave velocity in fluid	m/s
*C*_s_	Acoustic velocity in piezoelectric substrate	m/s
*λ*	Wavelength	m
*Zp*	Acoustic impedance of particle	Pa·s/m^3^
*Zm*	Acoustic impedance of surrounding medium	Pa·s/m^3^
*K*_*tr*_	Dimensionless coefficient	-
*R*_*p*_	Radius of the particle	nm
*R*_*b*_	Bubble radius	nm
*F*_PRF_	Pulse reputation force	Newton
*k*	Wave number	1/m
ρ_*l*_	Density of surrounding fluid	kg/m^3^
ρ_*p*_	Density of particle	kg/m^3^
*F*_*a*_	Axial force on particle	Newton
*F*_*d*_	Stokes force	Newton
*ϕ*	Acoustic contrast factor	dB
*P*_0_	Acoustic pressure amplitude	Pa
*x*	Axial distance of the particle from the pressure node	m
*V*_*p*_	Particle volume	m^3^
*v*	Relative velocity between the fluid and the particle	-
*u*	Fluid viscosity	m/s
β_*p*_	Particle compressibility	GPa/m^3^
β_*l*_	Fluid compressibility	GPa/m^3^
θ_*R*_	Rayleigh angle	-
*F*_SRF_	Surface radiation force	Newton
*d*	Distance between particles	m
*ω*	Angular frequency	Rad/s
*A*_*b*_	Amplitude of bubble oscillation	m

## Theory and mechanism of acoustofluidic separation

This section aims to introduce the various types of acoustic waves utilized, the principle behind acoustic excitation, and several crucial parameters that determine the migration of sorting targets. These parameters include the Rayleigh angle, Stokes force, and acoustic radiation force. The second part of this section aims to provide mechanisms of acoustofluidic separation.

### Acoustic waves

**Fig. 1:**
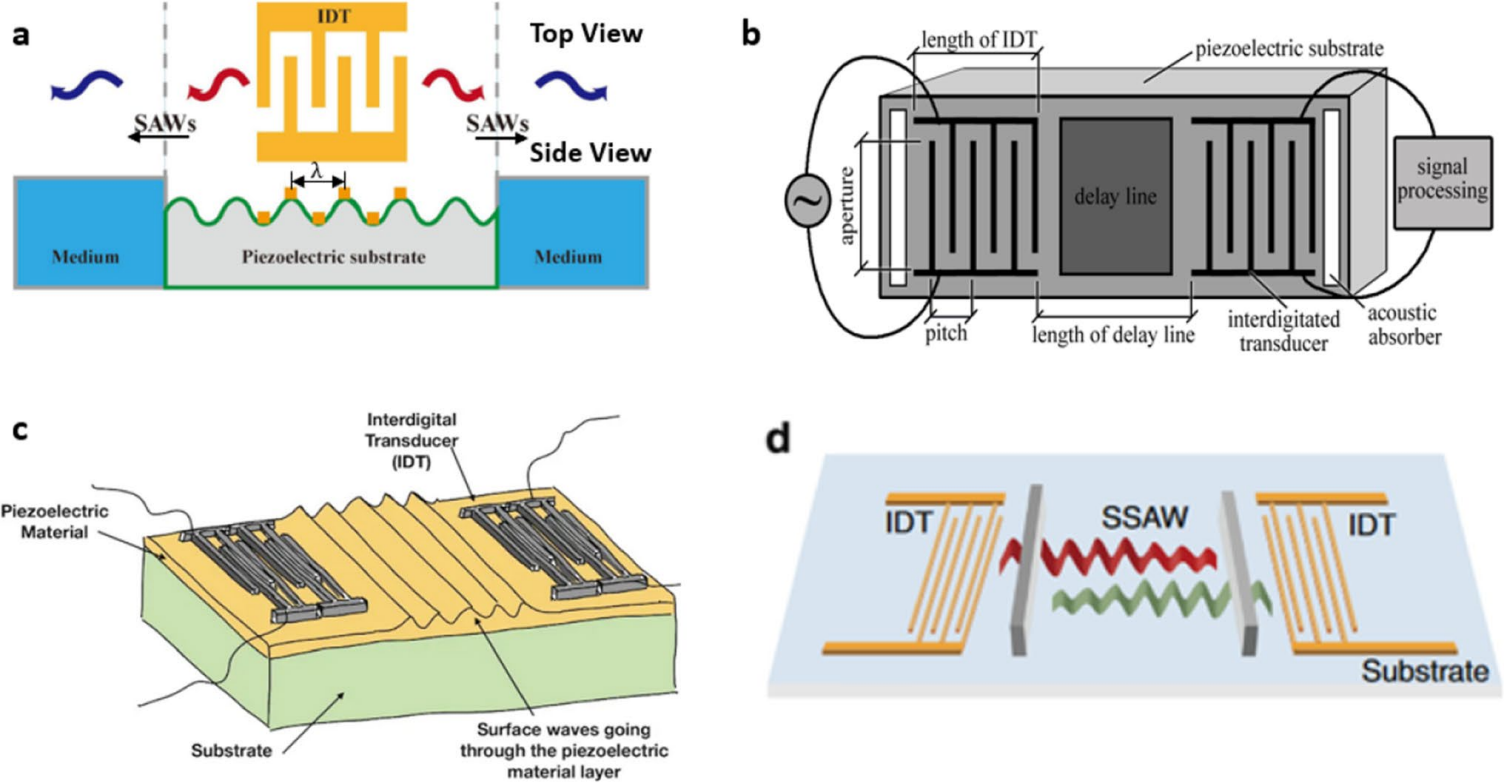
Exhibits multiple schematic diagrams, including **a** a surface acoustic wave generator (Jiang et al. 2021), **b** a schematic diagram of acoustic wave sensor (Zhou et al. 2011), **c** bulk acoustic wave filter (Qualcomm C, 2023), and **d** diagram of standing surface acoustic waves (Fan et al. 2022)

Mechanical waves known as acoustic waves are produced through the high-frequency vibration of piezoelectric materials such as quartz, lithium tantalite, or lithium niobate, when these materials are stimulated by alternating current (AC) (Mehmood et al. 2022). Acoustic waves are classified as—bulk acoustic waves (BAWs) and surface acoustic waves (SAWs)—based on whether the entire body or just the surface of the material undergoes vibration. Moreover, in the field of acoustics, traveling waves and standing waves are also distinguished from each other. Traveling waves move unidirectionally with uniform propagation, while standing waves are composite waves that propagate bilaterally (Maksymov et al. 2022; Singh et al. 2022). Standing waves that propagate within the microchannel and define the resonance chamber or cavity are known as BAWs (see Fig. 1b). Upon activation of the piezoelectric material, acoustic waves propagate into the microchannel via the solid-liquid interface. These waves resonate inside the channel only when the channel width is equal to an integer multiple of half-wavelength of the acoustic wave. BAWs emerge due to the reflection of acoustic waves from the channel wall (Ozcelik et al. 2022). Soft polymer materials like polydimethylsiloxane (PDMS) are not ideal for channel materials due to the formation of BAWs relying on channel wall reflection. On the other hand, materials like silicon and glass with superior acoustic properties are more appropriate for channel fabrication (Safaee et al. 2022; Karaman 2022). For the propagation of BAWs, the acoustic impedance of the substrate and the quality factor of the resonator are crucial factors to consider (Kvashnin et al. 2022; Xie et al. 2023). Significant attenuation of BAWs can result from both the inhomogeneity of the acoustic impedance of the piezoelectric substrate and the low-quality factor of the resonator (Xie et al. 2023; Alekseev et al. 2023). Based on differences in acoustic vibration modes and boundary conditions, they can be further classified into various types, including Lamb waves, Love waves, surface transverse waves, horizontal shear waves, leaky surface acoustic waves, Rayleigh waves, and electroacoustic waves (Zhang 2022; Ba Hashwan et al. 2023). By stimulating interdigitated transducers (IDTs) fabricated on a piezoelectric crystal, it is possible to produce vibrations on the surface of the material known as surface acoustic waves (SAWs). The wavelength of these SAWs (λ) is determined by the width and spacing between the fingers of the IDT.

Figure 1a depicts a typical SAW generator consisting of a substrate made of piezoelectric material and metal interdigital transducers (IDTs) deposited on it (McKibben et al. 2023). In Fig. 1a, the upper section illustrates an interdigital transducer (IDT) consisting of a series of metallic fingers. The characteristics of the generated surface acoustic wave (SAW) are determined by the structure of the IDT, including its bandwidth and directivity. By altering the number, spacing, and aperture (overlapping length) of the metallic fingers, one can modify the properties of the resulting SAW.

For instance, a focused IDT consists of pairs of annular electrodes that concentrate SAW energy to a small, localized focal point. On the other hand, a chirped IDT exhibits a gradient in the width of the electrode fingers along the SAW propagation direction, enabling it to generate SAWs across a wide frequency range. Meanwhile, a slanted finger IDT possesses a gradient in electrode finger width perpendicular to the SAW propagation direction, resulting in the generation of narrow SAW beams with varying frequency along the length of the fingers. The yellow dots in the wavy section of Fig. 1a represent the number of metallic fingers present in the IDT.

Figure 1b depicts the fundamental elements of a SAW sensor. It consists of a piezoelectric substrate that is capable of converting mechanical force into electrical charges and vice versa. Additionally, the sensor includes at least one interdigital transducer (IDT) that converts electromagnetic waves into acoustic waves and vice versa. Furthermore, the sensor encompasses a propagation area, commonly known as a delay line, which facilitates the transmission and propagation of the acoustic wave.

Figure 1c is showing the bulk acoustic wave filter. Filter technology plays a crucial role in the RF (radio frequency) signal chain as it enables signal selectivity. Both SAW and BAW filters rely on piezoelectric transduction to operate. SAW filters work by transducing an electrical signal into a piezoelectric material, causing it to vibrate at a specific natural frequency. These vibrations carry only a subset of frequencies from the input to the output transducer, effectively filtering out unwanted frequencies and returning the desired signals to the electrical domain. The signal is transduced laterally along the surface of the piezoelectric material between interdigital transducers. On the other hand, BAW filters consist of two main designs: FBAR (thin-film bulk acoustic wave resonator) and SMR (solidly mounted resonator). In an FBAR, there is a cavity beneath the support substrate, and careful design is required to minimize boundary leakage of the device. In contrast, the SMR incorporates Bragg layers as an acoustic reflector, reducing leakage within the substrate. Both designs aim to achieve efficient channel filtering using piezoelectric material vibrations.

Applying a sinusoidal AC signal to the IDTs causes subtle mechanical deformations to occur on the surface of the piezoelectric substrate. Consequently, mechanical SAW is produced and travels along the solid-air surface in the direction of deformation (Liu et al. 2023). The wavelength of SAWs is determined by the spacing and width between IDT fingers. To calculate the acoustic frequency, the following equation is used:

1 $$f=\frac{c}{\lambda }$$

where *C* is the speed of sound in the material, and λ is the wavelength of the sound.

Bulk acoustic wave (BAW), surface acoustic wave (SAW), and traveling acoustic wave (TSAW)

#### Bulk acoustic wave (BAW)

It operates within the bulk of a piezoelectric material, such as a crystal or a thin film. The waves travel through the thickness of the material. BAW devices typically consist of two electrodes sandwiching the piezoelectric material to generate and receive the waves. BAW filters offer excellent performance in terms of frequency stability, high power handling, and low insertion loss. They are commonly used in applications such as wireless communication systems, frequency control devices, and sensors.

#### Surface acoustic wave (SAW)

It propagates along the surface of a piezoelectric substrate, such as quartz or lithium niobate. The waves travel parallel to the surface, inducing particle motion primarily in the horizontal plane. SAW devices utilize interdigital transducers (IDTs) to generate and detect the waves. SAW filters offer high-frequency selectivity, low insertion loss, and compact size.

#### Traveling surface acoustic wave (TSAW)

It is a variant of SAW that combines both longitudinal and shear wave components. The waves propagate along the surface, inducing particle motion in both horizontal and vertical directions. TSAW devices often employ metal gratings or arrays to generate and receive the waves. TSAW filters provide enhanced frequency selectivity, improved performance, and reduced insertion loss compared to traditional SAW filters. They are suitable for applications such as wireless communication systems and high-frequency signal processing.

In summary, BAW operates within the bulk of a material, SAW propagates along the surface, and TSAW combines both longitudinal and shear wave components for improved performance.

### Acoustic radiation forces (ARFs)

The ARF, or acoustic radiation force, is generated by the nonlinear propagation of sound waves in the fluid medium, resulting in an acoustic pressure gradient. This gradient exerts a force on particles present in the medium, causing them to experience a displacement or motion. The three ARFs (acoustic radiation forces) can be defined as follows:

1. Primary acoustic radiation force (PRF): This force arises from the interaction between the acoustic wave and the particles in the medium. It can be further divided into two components.

- Axial component (*F*_*a*_): This component acts along the direction of sound propagation and is responsible for particle displacement in that direction.
- Transverse component (Ft): This component acts perpendicular to the direction of sound propagation and causes particles to move closer together.

2. Secondary acoustic radiation force (SRF): This force emerges from the interaction between particles and other scatterers present in the medium, such as bubbles or other particles. It can be attractive or repulsive, depending on the configuration and characteristics of the particles or scatterers involved.

The interrelationships between these three ARFs are as follows:

- The PRF arises directly from the acoustic wave and is primarily responsible for particle displacement due to the acoustic pressure gradient generated by the wave.
- The SRF emerges from the scattered sound waves by the particles or scatterers, and it can enhance or counteract the effects of the PRF on particle motion.
- Both PRF and SRF contribute to the overall movement and aggregation of particles in an acoustic field, but they arise from different mechanisms and interactions within the medium.

It is important to note that the specific calculations and formulas for these forces may vary depending on the experimental setup, particle properties, and medium characteristics.

**Fig. 2:**
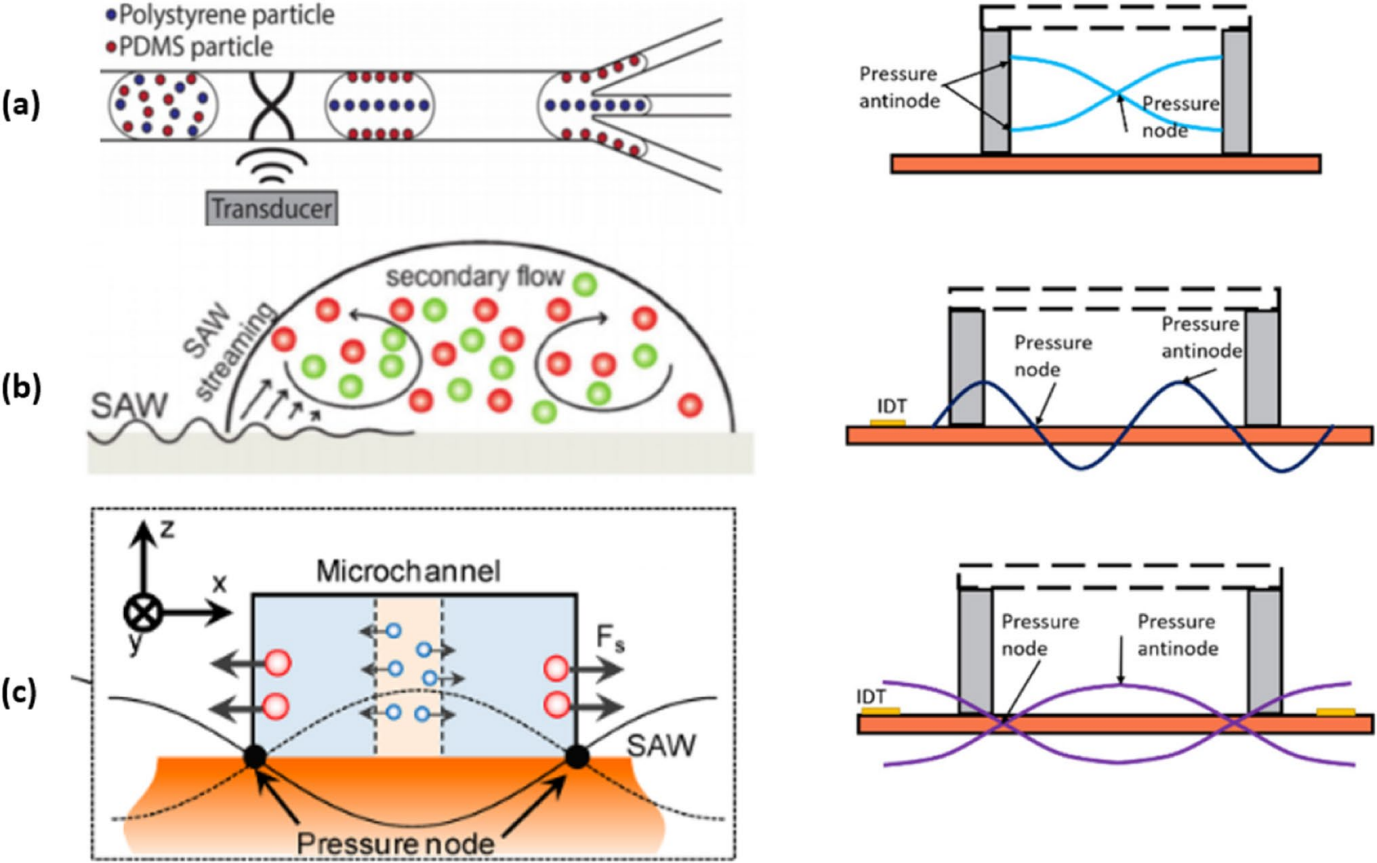
The different types of acoustic waves, including bulk acoustic wave (BAW), standing surface acoustic wave (SSAW), and traveling surface acoustic wave (TSAW) (Ai et al. 2013). On the left side, there are three illustrations. **a** the BAW-based separation technique for polystyrene and polydimethylsiloxane (PDMS) particles with varying acoustic contrast factors. **b** The TSAW-induced acoustic streaming method for density-based separation. With TSAW actuation, lighter particles are dispersed towards the periphery, while more dense particles accumulate in the center. **c** The cross-section visual representation of SSAW-induced particle separation. Pressure nodes are positioned at the two sidewalls, where the two particles are mutually attracted to each other (Gao et al. 2020)

The emergence of ARF acting on particles is due to the nonlinear propagation of sound within the fluid medium, which generates an acoustic pressure gradient. As illustrated in Fig. 2, the traveling and standing surface acoustic waves generate acoustic streaming (TSAW) effects that influence the particles and cells within the medium (Mitchell 2022).

Within TSAWs, the paths of particles are influenced by the balance between the drag force induced by acoustic streaming and the acoustic radiation force ARF. Skowronek et al. (2013) introduced a dimensionless coefficient (*K*_*tr*_) to determine the dominant force.

2 $${K}_{tr}=\frac{2\pi {R}_p}{\lambda }$$

where *R*_*p*_ is the radius of the particle and *λ* is the sound wavelength.

If *K*_*tr*_ < 1, the acoustic streaming-induced drag force will be the dominant force, causing the particle to move towards the acoustic streaming vortex. On the other hand, if *K*_*tr*_ > 1, the acoustic radiation force (ARF) will be the primary force that propels the particle, causing it to move away from the IDT. King et al. (1943) formulated the PRF (pulse reputation force) acting on particles in a TSAW using the following equation:

3 $${F}_{\textrm{PRF}}=2\pi {\rho}_l{\left|A\right|}^2{\left(k{R}_p\right)}^6\ \frac{9+2\left(1+{\lambda}_p\right)}{9\left(2+{\lambda}_p\right)}$$

Were ${\lambda}_p=\frac{\rho_l}{\rho_p}$where *A* denotes the complex amplitude of velocity potential, *k* represents the wavenumber of acoustic radiation, *R*_*p*_ represents the radius of the particle, and *ρ*_*l*_ and *ρ*_*p*_ are the densities of the surrounding fluid and the particle, respectively.

In SSAW (standing surface acoustic waves), the primary acoustic radiation force (PRF) can be further divided into two components: the axial component (*F*_*a*_) and the transverse component (*F*_*t*_). The axial component, (*F*_*a*_), is calculated using the following equation:

4 $${F}_a=\left(\frac{\pi {P}_0^2{V}_p{\beta}_l}{2\lambda}\right)\phi \left(\beta, \rho \right)\sin (kx)$$

were $$\phi \left(\beta, \rho \right)=\frac{5{\rho}_p-2{\rho}_l}{2{\rho}_p+{\rho}_l}-\frac{\beta_p}{\beta_l}$$.

The axial force exerted on particles by *F*_*a*_ is influenced by several factors. These includes the following:

- The acoustic contrast factor (*ϕ*)
- Acoustic pressure amplitude (*P*_0_)
- Axial distance of the particle from the pressure node (*x*)
- Particle volume (*V*_*p*_) and compressibility (*β*_*p*_)
- Fluid compressibility (*β*_*l*_)

Particles can be driven towards pressure nodes or antinodes through *F*_*a*_, with the acoustic contrast factor (*ϕ*) playing a crucial role in determining the direction of particle motion. When *ϕ* is greater than zero, particles move towards the pressure nodes. On the other hand, when *ϕ* is less than zero, particles move towards the pressure antinodes. When particles are directed towards the nodal plane, the axial force becomes negligible, and the transverse pressure force (*Ft*) becomes dominant; this is calculated as follows.

5 $${F}_t=3{d}_p^3\frac{\rho_p-{\rho}_l}{2{\rho}_p+{\rho}_l}\nabla <E>$$

where ∇<*E*> denotes the acoustic energy gradient, <> represents the time average, and *d*_*p*_ represents the distances between particles.

The transverse pressure force in SSAW systems acts to push particles closer to each other. As the distance between the particles decreases, the force gradually becomes weaker. Ultimately, the particles tend to aggregate together at either the pressure node or the pressure antinode of the standing surface acoustic wave. This phenomenon occurs due to the spatial variation of the acoustic pressure field, which leads to the attraction and gathering of particles in specific regions of the wave.

While the PRF mainly influences the migration of a single particle, the SRF becomes important when multiple cells or particles aggregate. When the distance between particles is small, the trajectory of the particles is significantly influenced by the SRF (Blackburn et al. 1991). Silva and Bruus (2014) showed that when the acoustic wavelength is much greater than the particle size, two particles in close proximity can either attract or repel each other perpendicular to the wave propagation within the Rayleigh limit. The interparticle ARF is proportionate to the size of particles (Meng et al. 2019). The SRF equation was derived by Weiser et al. (1984) for two particles with identical acoustic properties and radius in the Rayleigh limit as

6 $${F}_{\textrm{SRF}(x)}=4{r}^6\left[\frac{{\left({\rho}_p-{\rho}_l\right)}^2\left(3\;{\cos}^2\theta -1\right)}{6{\rho}_l{d}^4\ }{v}^2(x)-\frac{\omega^2{\rho}_l{\left({k}_p-{k}_l\right)}^2}{9{d}^2}{p}^2(x)\right]$$

In the given context, the variables *v*(*x*) and *p*(*x*) represent the particle velocity and acoustic pressure, respectively. The angle θ represents the angle between the centerline that connects two particles and the direction of acoustic wave propagation. The angular frequency of the sound wave is denoted by ω, and *d* represents the distance between the centers of the two particles.

When considering a particle in close proximity to a bubble, the secondary acoustic radiation force (SRF) can be calculated using the following equation:

7 $${F}_{\textrm{SRF}}=4\pi {\rho}_l\ \frac{\rho_p-2{\rho}_l}{\rho_l+2{\rho}_p}-\frac{R_b^4{R}_P^3}{d^5}{\omega}^2{A}_b^2$$

The direction of the force acting on a particle is determined by several factors, including the densities of the surrounding fluid (*ρ*_*l*_) and particles (*ρ*_*p*_), the distance between the particle and the bubble (*d*), the angular frequency (*ω*), the amplitude of bubble oscillation (*A*_*b*_), and the radii of the bubble (*R*_*b*_) and particle (*R*_*p*_). The nature of the force—whether it is attractive or repulsive—is determined based on the specific values of *ρ*_*l*_ and *ρ*_*p*_.

### Stokes force

Acoustic streaming refers to a stable flow within a fluid that is induced by the absorption of high-frequency and high-amplitude acoustic waves. The generation of acoustic streaming is attributed to the fluid’s viscous attenuation characteristic of the fluid. The particles and cells within acoustic streaming experience a resistance force, known as the Stokes force (*F*_*d*_) calculated from (Garrett and Garrett 2020, Pralle et al. 1998):

8 $${F}_d=6\pi u{R}_pv$$

In the formula, *u* represents the fluid viscosity, *v* denotes the relative velocity between the fluid and particles, and *R*_*p*_ represents the radius of particle.

### Rayleigh angle

Surface acoustic waves (SAWs) experience an exponential decline in amplitude during their transmission through the channel wall. The acoustic waves that persist effectuate propagation along the substrate until the acoustic streaming coupling phenomenon occurs. Consequently, the formation of “leakage surface acoustic waves” happens in the microchannel (Gutiérrez Ramos 2018). The propagation velocities of SAWs in the fluid and substrate exhibit dissimilarities owing to the variation in their viscosity. As a result, the acoustic waves undergo refraction at the fluid-solid interface and penetrate the fluid medium at a distinct angle which is called Rayleigh angle (Andersen and Au 1999).

9 $${\theta}_R=\arcsin \left(\frac{C_1}{C_s}\right)$$

*C*_1_ and *C*_*s*_ represent the acoustic wave velocities in the fluid and piezoelectric substrate, respectively. The acoustic speed of the piezoelectric material varies with respect to different tangential directions, as influenced by its anisotropic nature. The tangential direction of the piezoelectric substrate governs the Rayleigh angle. This angle triggers the “anechoic corner effect, where the upper corner of the microchannel experiences insufficient ARF, thereby having minimal impact on the particles situated there (Wong et al. 2019).

Bulk acoustic waves have emerged as a valuable tool for microfluidic separations due to their advantageous features such as flexible transducer placement and simple and versatile setups (Zhao et al. 2023). In a BAW-based microfluidic device (Fig. 2a), bulk acoustic standing waves are generated within a microchannel that lies between two parallel opposite walls. Through the utilization of a piezoelectric transducer, BAWs can be induced in a fluid-filled microchannel, leading to resonance when encountering acoustically contrasting materials like silicon, polydimethylsiloxane (PDMS), or glass (Ng 2020). Compared to SSAW-based separation devices (Fig. 2c), BAW-based systems typically employ lower frequencies and longer wavelengths. This characteristic enables them to handle larger particles effectively. By applying BAW to the system, the acoustic streaming-induced drag force selectively transported smaller particles to the desired location, while larger particles were predominantly influenced by the acoustic radiation force. TSAW-based separation (Fig. 2b) has been extensively utilized for sorting microparticles of different sizes, including polystyrene (PS) particles, fused silica (FS) particles, and polymethyl methacrylate (PMMA) particles (Destgeer and Sung 2015, Liu et al. 2023).

### Acoustic impedance

Acoustic impedance (*Z*) of a medium is determined by multiplying the density (*ρ*) of the medium and the speed of sound (*c*) within the specific medium. Mathematically, it can be expressed as follows (Hossein 2019):

10 $$z=\rho c$$

The density (*ρ*) of a material refers to how much mass it possesses per unit volume. It quantifies the compactness of the material. Higher density materials have more closely packed particles, while lower density materials have more dispersed particles.

Acoustic impedance (*Z*) quantifies the resistance that sound waves encounter when propagating through a medium. It is important to note that the acoustic impedance of different media can vary significantly, impacting the transmission and reflection of sound waves at the interface between different materials. For example, if sound waves pass from a medium with a low impedance to a medium with a high impedance, a significant portion of the sound energy may be reflected back rather than transmitted through. According to Eq. 10, an increase in the acoustic impedance of the medium will result in an increase in the sound speed, assuming that the density remains constant. Similarly, a decrease in the sound speed will result in a decrease in the acoustic impedance.

It is important to note that the relationship between sound speed and density is influenced by various factors, such as temperature, pressure, and the composition of the medium (Marsh et al. 2002). Additionally, different materials can have distinct relationships between sound speed and density, so it may not be a universal relationship across all substances (Dalmont 2001).

The relationship between acoustic impedance, density, and bulk modulus (B) can be given as follows:

11 $$z=\sqrt{\rho B}$$

where *B* is the bulk modulus, and ρ is the density of the medium.

Compressibility, on the other hand, is a measure of how easily a material can be compressed or deformed under the application of external forces. It is typically represented by the bulk modulus (*B*), which relates the stress applied to a material to the resulting strain. Low compressibility indicates that a material is stiff and resistant to deformation, while high compressibility suggests that a material can be easily compressed or deformed (Karthick et al. 2018).

The correspondence between acoustic impedance and the mechanical properties of an object, such as density and compressibility, is crucial in acoustofluidic methods, particularly in particle separation techniques like exosome separation. By exploiting differences in the mechanical properties of various substances or particles, acoustic waves can induce displacement or motion of particles within a fluid medium. The particles experience different acoustic radiation forces based on their acoustic impedance, density, and compressibility. This allows for selective separation and manipulation of particles based on their mechanical properties. When sound waves travel from one medium to another, such as from air to water, the acoustic impedance mismatch between the two mediums can result in the reflection or transmission of sound waves. If the acoustic impedance of the two mediums is similar, such as in the case of air and helium, there is minimal reflection, and the sound waves are transmitted without significant energy loss. However, when there is a significant acoustic impedance mismatch, such as when sound waves travel from air to water, a portion of the energy is reflected back and not transmitted. This can result in a decrease in sound intensity in the fluid. Furthermore, the acoustic impedance of a fluid also affects the overall propagation of sound waves through it. In a fluid with a higher acoustic impedance, the speed of sound is generally lower, leading to a decrease in the sound intensity. Conversely, in a fluid with a lower acoustic impedance, the speed of sound is generally higher, resulting in an increase in the sound intensity (Ozcelik et al. 2022). The acoustic impedance and sound speed of different materials in relation to their physiochemical properties are given in Table 3.

**Table 3:** Acoustic impedance and sound speed of different materials in relation to their densities

Material	Density ρ(kg.m^−3^)	Speed *c*(m.s^−1^)	Acoustic impedance *Z*(kg.m^−2^).s^−1^) × 10^6^
Water	1000	1480	1.5
Blood	1060	1570	1.62
Bone	1380–1810	4080	3.75–7.38
Brain	1030	1558	1.55–1.66
Fat	920	1450	1.35
Kidney	1060	1560	1.62
Liver	400	650	1.64–1.68
Liver	1070	1584	0.26
Muscle	1070	1584	1.65–1.74
Spleen	1060	1566	1.65–1.67

### Mechanisms of acoustofluidic separation

Acoustofluidic separations refer to the use of acoustic waves to manipulate and separate particles or cells in a fluid medium. This technology enables the precise and gentle separation of cells based on their physical properties, such as size, density, and compressibility (Charan et al. 2023). Acoustofluidic separations have been used widely in biological and medical research, as well as in the development of diagnostic and therapeutics devices. They have several advantages over traditional separation methods, including high throughput, low cost, and compatibility with sensitive biological samples (Devendran and Neild 2022, Rasouli 2022).

**Fig. 3:**
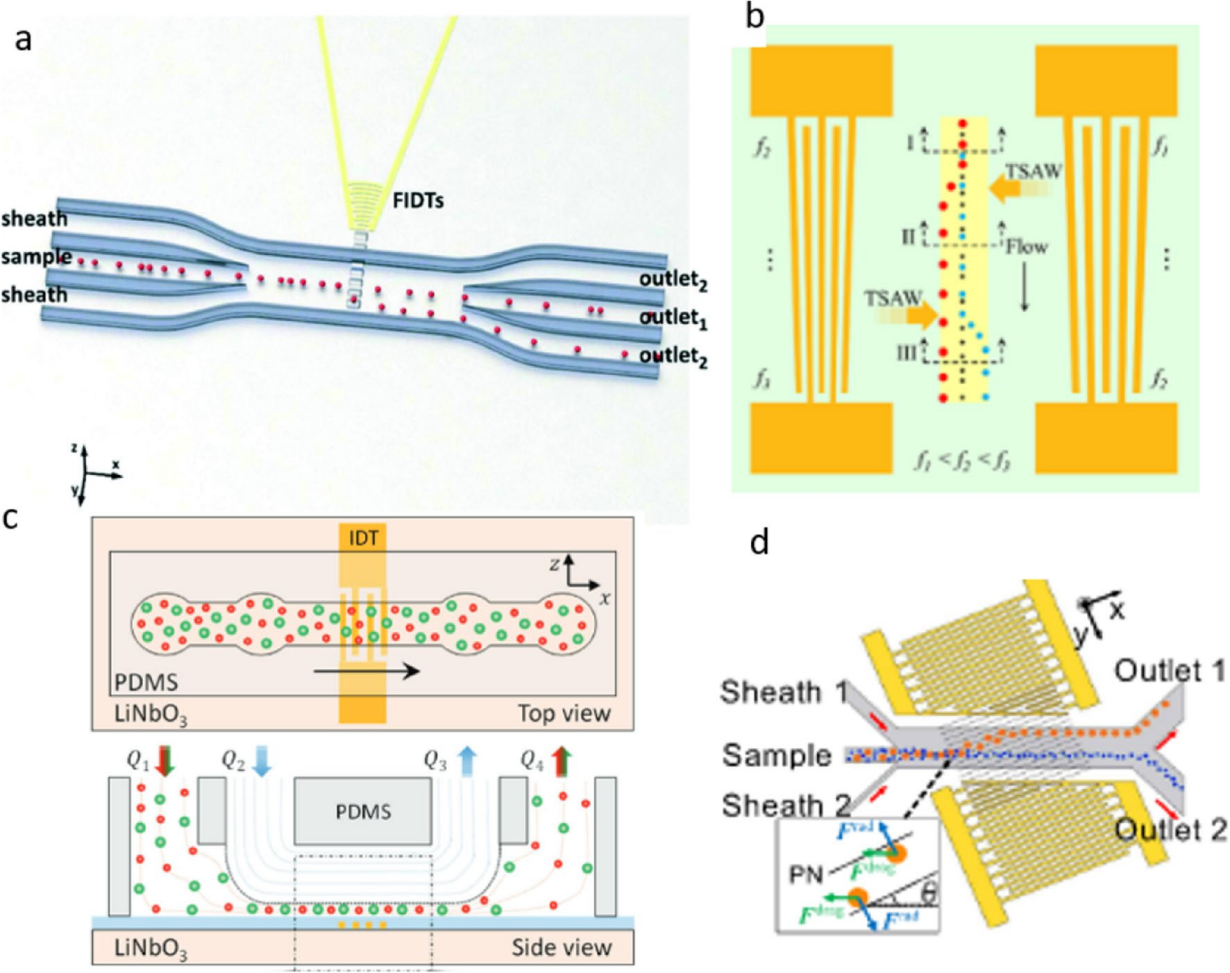
**a** For particle separation, interdigital transducers that focused parallel to the microchannel were positioned to generate surface acoustic waves with high energy density (Collins et al. 2016). **b** In reference (Destgeer et al. 2015), particle separation was achieved using a pair of slanted interdigitated transducers that were positioned on opposite sides of the microchannel and activated with different frequency signals. **c** Vertical migration separation of polystyrene particles of distinct sizes was accomplished using an interdigital transducer positioned beneath the microchannel (Ahmed et al. 2018). **d** Cell deflection was improved in the microchannel by using a couple of tilted-angle interdigital transducers (Wu et al. 2021)

The acoustic waves create pressure variations within the fluid that give rise to acoustic radiation forces (Tang and Huang, 2022), acoustic streaming forces (Hellemann et al. 2022), and acoustic standing waves (Pavlic et al. 2022). These forces can be modulated by manipulating the frequency, amplitude, and phase of the acoustic waves to selectively act on particular particles or cells based on their physical or mechanical properties, such as size, density, compressibility, or deformability (Dholakia et al. 2020; Toftul et al. 2019). The use of piezoelectric transducers enables the precise control and manipulation of these forces, making acoustofluidic separation a highly efficient and versatile technique (Ozcelik and Huang 2021; Hossein and Wang 2020; Hossein et al. 2021; Hossein et al. 2022). Surface acoustic waves (SAW) occur when the electric signal is applied to the edges of the piezoelectric material, inducing a wave that propagates along its surface (Huang et al. 2021). SAW modes are typically used to generate acoustic forces at the microscale, where their small wavelength enables higher resolution and greater sensitivity compared to BAW modes (Rufo et al. 2022). SAW can be further classified based on the direction of the wave propagation and the orientation of the piezoelectric substrate, with the most common modes being Rayleigh, Sezawa, and Love waves (Ba Hashwan et al. 2023). Rayleigh waves, which propagate in a direction perpendicular to the surface, are widely used in microfluidic systems due to their relatively low attenuation and high sensitivity to surface confinement. Rayleigh waves are particularly useful for fluid and particle manipulation, as they can induce both acoustic radiation and streaming forces to act on particles, while minimizing the risk of damage or cell lysis. Sezawa waves, which propagate in a direction parallel to the surface, are less commonly employed, but they can provide enhanced sensitivity to mechanical and electrical properties of inclusions within fluids (Liu et al. 2023). Love waves, which are a combination of Rayleigh and Sezawa waves, are also used in microfluidic systems for their high sensitivity to changes in the surface properties of the substrate, particularly for biosensing applications (Song et al. 2022). By selecting a specific acoustic mode and tuning its parameters, acoustofluidic separation can achieve precise and selective manipulation of particles and cells in a fluid sample, making it a powerful tool for a range of biomedical and biotechnological applications (Rasouli et al. 2023). The separation based on size can be achieved with various designs and positions of interdigital transducers (see Fig. 3).

Figure 3a shows focused interdigital transducers (FIDTs) placed next to a microchannel to generate high-energy-density traveling surface acoustic waves (SAWs) for particle separation. The schematic illustration in Fig. 3a introduced by Collins et al. (2016) demonstrates the device design, a sheath configuration was utilized to hydrodynamically focus the sample before the FIDTs. This force field enables single-particle level sorting, and due to the high-frequency nature of the device, even particles as small as 2 μm can be separated. This system has potential applications in various fields such as diluting particle/cell mixtures on demand and selective cell sorting.

 Figure 3b illustrates the use of two slanted interdigitated transducers (SIDTs) with different frequencies in a miniaturized acoustofluidic device. This device enables size-selective separation and medium exchange around polystyrene particles in a continuous, label-free, and contactless manner. The SIDTs generate tunable traveling surface acoustic waves (TSAWs) that create an anechoic corner inside a microchannel. By using different frequencies, larger particles are laterally deflected to the top-left corner, while medium-sized particles are deflected to the right, leaving smaller particles in the middle of the microchannel. This achieves particle separation. Additionally, the anechoic corner can be used to deflect a particle not originally present there, enabling medium exchange. The study successfully demonstrates three-way separation of polystyrene particles with different diameters and showcases multimedium exchange around specific particle sizes.

Another device for separating particles as shown in Fig. 3c consists of a microchannel made of polydimethylsiloxane (PDMS) attached to a piezoelectric substrate. A mixture of two different-sized particles is injected through one inlet, while a sheath fluid is introduced through another inlet. The sheath fluid helps create a double-layered flow and focuses the particles close to the bottom of the microchannel. The fluid is pumped out through one outlet, while the remaining fluid is collected through another outlet. When the power is off, the particles flow through the lower streamlines and can be collected together. However, when an AC signal is applied to the device, it generates an acoustic wave that affects the larger particles, causing them to move against the flow direction and be collected through the first outlet. The smaller particles continue to flow unaffected and are collected through the second outlet. This allows for continuous separation of particles based on their size difference (Ahmed et al. 2018).

A recent device for continuous separation of particles with different sizes by TSAW (traveling surface acoustic wave) is shown in Fig. 3d. The device utilizes the vertical component of the ARF (acoustic radiation force) to push selected particles upwards in the microchannel. The horizontal component of the ARF is used to slow down separated particles laterally, giving them more time for vertical migration and improving separation efficiency. Wu et al. (2021) demonstrated the successful separation of 4.8 μm Polystyrene particles from 2.0 and 3.2 μm particles with high purity (>99%) and recovery.

To overcome limitations of traditional acoustofluidic sorting devices, where IDTs (interdigital transducers) are placed parallel to the microchannel, a new layout was introduced (Wang et al. 2021), where the IDTs are positioned at an angle to the channel, allowing for increased particle deflection. Two types of devices were developed using this approach—tilted-angle traveling surface acoustic wave (taTSAW) devices and tilted-angle standing surface acoustic wave (taSSAW) devices.

For taTSAW devices, the IDTs are placed on one side of the channel at a tilted angle to generate a traveling SAW. Ahmed et al. (2018) developed a taTSAW device that achieved sheathless focusing and separation of microparticles in continuous flow. Two IDTs were set at angles of 210° and 150° relative to the principal axis of the substrate wafer. The first IDT pushed all particles to one side of the microchannel using a frequency of 194 MHz, and the second IDT successfully separated 4.8 μm fluorescent PS particles from 3.2 μm particles with a purity above 99% using a frequency of 136 MHz.

BAW devices can be designed with variety of geometries, including straight, curved or spiral channels, to optimize the acoustic fields and enhance the separation efficiency (Shiri et al. 2022, Mahboubidoust et al. 2023). In addition, BAW devices can be integrated with other microfluidic components, such as pumps and valves, to create complex microfluidic systems for sample preparation and analysis. For SAW-based devices, the microfluidic channel is typically made of materials with high acoustic impedance, such as polymers or thermoplastics (Stoukatch et al. 2022, Gharib et al. 2022).

Acoustic waves are reflected perfectly by the channel walls due to the significant impedance mismatch between the channel material and fluid medium (Ren et al. 2022). By adjusting the width or depth of the channel to match multiples of the acoustic wavelength, an acoustic resonator is created, allowing for the formation of a standing acoustic wave field using two pairs of interdigital transducers (IDTs) in SAW-based devices (Mazalan et al. 2021). The interference of counter-propagating SAWs generates a standing SAW field that can be transmitted through a microfluidic channel to excite longitudinal acoustic waves in the liquid and create nodes and antinodes of pressure (Richard et al. 2019). These periodic pressure fluctuations induce forces that are utilized for particle and cell separations. Both standing and traveling SAWs can be used to achieve separation, with the latter utilizing differential effects to separate various particles and cells (Lemma 2022). Primary acoustic radiation forces move particles towards pressure nodes or antinodes in the acoustic field, while secondary forces drive particle aggregation or segregation.

**Fig. 4:**
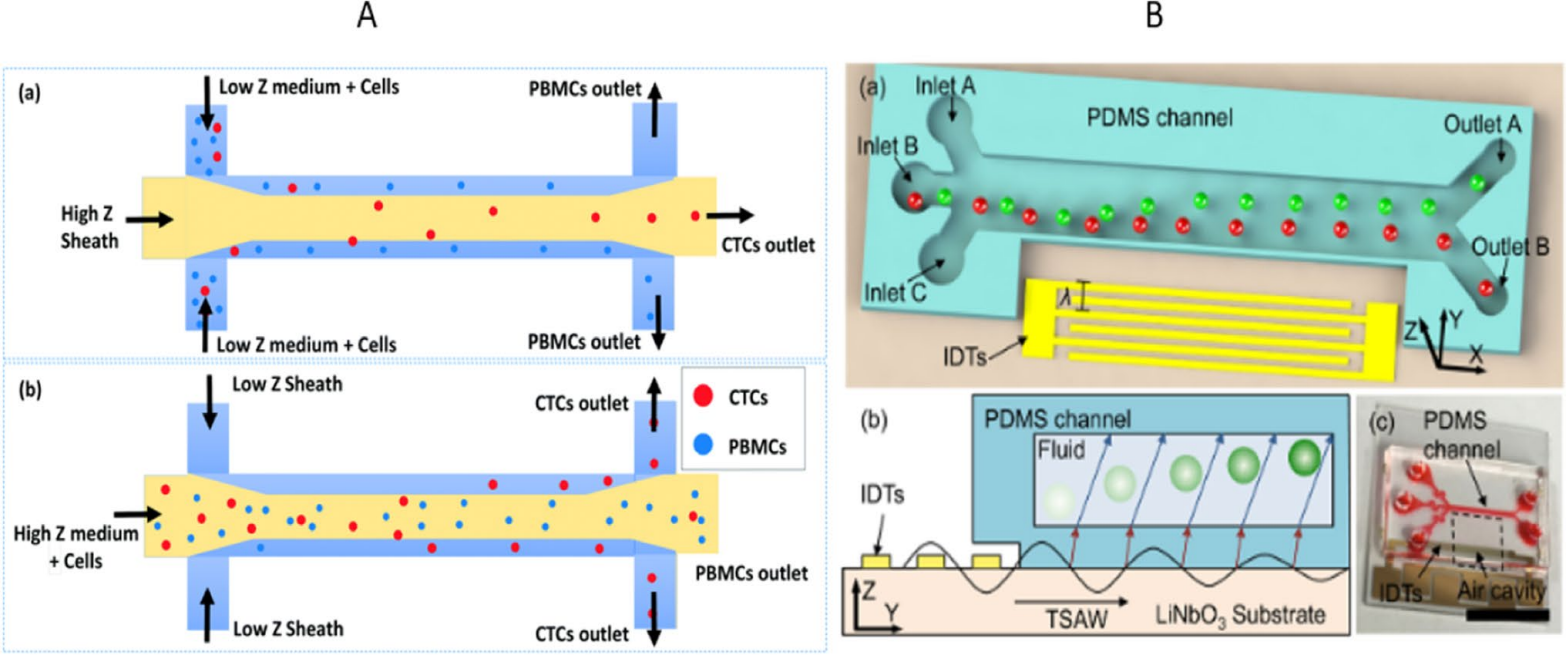
**A** The acoustic impedance contrast is utilized to isolate HeLa and MDA-MB-231 cells from peripheral blood mononuclear cells (Karthick et al. 2018). **B** A traveling surface acoustic wave device is utilized to differentiate polystyrene and polymethyl methacrylate particles with identical diameters, based on variations in particle density and sound propagation speed (Ma et al. 2016b)

Figure 4A is describing a method for efficiently isolating circulating tumor cells (CTCs) from peripheral blood mononuclear cells (PBMCs) using acoustophoresis. By creating an inhomogeneous liquid flow configuration, acoustic standing waves relocate high impedance liquids to the center of the channel and low impedance liquids to the sides, creating a stable configuration. For CTCs with higher impedance than PBMCs, they are suspended in a matching medium and passed through side inlets while higher impedance sheath fluid is passed through the center inlet. This causes the CTCs to migrate laterally towards the center of the channel, while PBMCs continue along the streamlines in the matching medium. This is called positive impedance sorting. For CTCs with lower impedance than PBMCs, the configuration is reversed. CTCs are suspended in the matching medium and passed through the center inlet, while lower impedance sheath fluid is passed through the side inlets. This causes the CTCs to migrate laterally towards the sides of the channel. PBMCs continue along the streamlines in the matching medium. This is called negative impedance sorting. If the impedance of CTCs overlaps with that of PBMCs, a size-based sorting technique can be used instead (Karthick et al. 2018).

Figure 4B shows the schematic and photograph of an acoustophoretic microfluidic system used for particle separation based on mechanical properties. The system consists of a transducer with interdigital transducers (IDTs) on a lithium niobate (LN) substrate. The IDTs are fabricated using a lift-off technique, and the width of the electrode elements is equal to 1/4 of the acoustic wavelength. The transducer generates a traveling surface acoustic wave (TSAW) that couples into a disposable polydimethylsiloxane (PDMS) channel. The channel has three inlets and two outlets, and particles flowing through the channel are transferred in different distances based on their mechanical properties and collected in different outlets. The PDMS channel can be easily peeled off and discarded while the TSAW device is retained for reuse. When an AC signal is applied to the IDTs, the TSAW is generated and transfers the desired particle population into different outlets.

In most current SAW devices, the microchannel layers and IDT layers are permanently fused together. (Chen et al. 2022). Although single-use devices are suitable for many biomedical applications, the high cost of fabrication of IDTs results in high test expenses (Witek et al. 2019). A disposable separation device based on TSAW was developed by Ma et al. (2016a), which was capable of effectively segregating PS particles measuring 10 and 15 μm in size.

## Separation methods based on nonsize properties

While size-based separation techniques have proved highly effective at separating varying-sized particles or cells, they are incapable of isolating particles of similar sizes or those that differ only slightly in size (Li et al. 2013). Acoustic impedance (Z), which is directly connected to a material’s density and sound speed, has been identified as a suitable property for sorting cells or particles. It has been demonstrated that under specific circumstances, the impedance difference between the particle and medium can determine the direction of particle migration (Levi and Aurbach 1997). If the acoustic impedance of particles is higher than that of the surrounding medium (*Zp* > *Zm*), the particles will move toward the pressure node. Conversely, if the impedance is lower than that of the medium (*Zp* < *Zm*), they will move toward the pressure antinode. When there is no impedance variation between the particle and medium, acoustic streaming is the only force experienced by the particles.

### Applications of acoustofluidic separation

Acoustofluidic separation has found extensive use in many applications, ranging from the isolation of rare circulating biomarkers to the differential focusing and separation of nanoparticles. The applications of acoustofluidic separations include the following:

1. Medical diagnosis and research: Acoustofluidic separations can be used for the separation of cells and biomolecules of different sizes and densities, which is useful for medical diagnosis and research (Li and Huang 2018).
2. Drug discovery: Acoustofluidic separations can be used for high-throughput drug discovery by separating target cells or molecules from a complex mixture (Nasiri et al. 2020).
3. Environmental monitoring: Acoustofluidic separations can be used for the separation of microorganisms and pollutants in environmental monitoring (Akiyama et al. 2020).
4. Food processing: Acoustofluidic separations can be used for the separation and purification of food ingredients, such as proteins and enzymes (Xie et al. 2020).
5. Industrial production: Acoustofluidic separations can be used for the separation and purification of various industrial materials, including chemicals, metals, and nanoparticles (Xie et al. 2020).
6. Water treatment: Acoustofluidic separations can be used for the removal of particles and impurities from water, such as bacteria, viruses, and microplastics (Chen et al. 2020).

## Separation of blood components

Separation of different blood components is crucial for diagnostics, as abnormal levels of each component may indicate various disease states (Greening et al. 2010). In therapeutic applications, transfusions of specific components can be used to address deficiencies. The purity and viability of separated cells are essential for accurate diagnostics and effective therapy (Plouffe et al. 2014). The primary components of blood include red blood cells (RBCs, 6–8 μm in diameter), white blood cells (WBCs, 12–15 μm in diameter), platelets (1–5 μm in diameter), and plasma. RBCs are the most abundant cells in the blood, with approximately 4–6 million cells per microliter (Fukushima et al. 2011). There are typically 4500 to 11,000 WBCs and 150,000 to 450,000 platelets per microliter of blood. Plasma, the liquid part of blood, contains various proteins, antibodies, and molecules. Each of these blood components has its unique functions and can serve as targets for diagnostic and therapeutic purposes (Ansari et al. 2018).

Conventional centrifugation is the method of choice for separating blood components, where blood is spun under a 3000x g force, resulting in three fractions: plasma, a buffy coat containing WBCs and platelets, and RBCs (Rutkowski 2008). Filtration is also used in some cases. However, centrifugation and filtration-based technologies are bulky and unsuitable for point-of-care applications. In addition, their efficiency and biocompatibility are limited (Yang et al. 2020). Acoustofluidic separation technologies have been demonstrated to continuously and biocompatibly separate blood components (Wu 2018). The processing acoustofluidic technologies have been found effective in the separation of blood components, including platelets, RBCs, and WBCs (Antfolk and Laurell 2019). Acoustofluidic separation has also been used to separate lipid particles carrying the risk of clogging in blood circulation (Ding et al. 2021).

Acoustofluidic separation techniques have demonstrated efficacy in various blood component separation applications, with some studies showing their biocompatibility in terms of low levels of platelet activation and preserving cell functions (Gu et al. 2019). Despite significant progress over the past decade, acoustofluidic-based blood component separation still has limitations. One of the primary drawbacks is the low throughput, typically in the μL/min range (Xie et al. 2019b). The medical technology apheresis is FDA-approved and widely used for the treatment of many diseases. Apheresis requires high-throughput processing of blood (30–80 mL/min) in a biocompatible manner, while simultaneously returning some blood components back into circulation. Unfortunately, current acoustofluidic separation techniques do not have the high throughput required for apheresis (Zhou and Papautsky 2020). Additionally, some acoustofluidic techniques require the modification of the carrier medium, making it challenging to return the blood components back into the body. Therefore, it is crucial to improve the throughput and precision of acoustofluidic techniques and avoid the use of undesirable carrier media to increase their clinical relevance (Wu 2018).

## Separation of bioparticles

### Separation of viruses

Viruses consist of genetic material enveloped by a protein coat and are typically small, ranging from 20 to 400 nm in diameter (Gelderblom 1996). They can invade host cells and result in illnesses like AIDS, hepatitis, and COVID-19 (Liu et al. 2020a). Currently, virus detection is considered successful through polymerase chain reaction (PCR) and enzyme-linked immunosorbent assays (ELISAs). Although both methods accurately detect viruses, they both require extensive time for detection, sophisticated equipment, and expert operation, making them not always ideal choices (De Paula and Fonseca 2004). Thus, it is crucial to explore novel approaches for swift and accurate virus detection and isolation. The use of acoustofluidic technology in virus isolation has been researched extensively (Xie et al. 2020). Since viruses are too small to be affected by ARFs, the process involves concentrating viruses by extracting them from a virus-cell mixture using ARF. In one example, Jung et al. (2021) exhibited a microfluidic BAW-based device with the ability to isolate *Saccharomyces cerevisiae* (*S. cerevisiae*) and MS2 bacteriophage. The H-filter device received the sample mixture and deionized water through the two inlets, forming a standing wave with a pressure node locating at the channel’s center. The ARF propelled the larger *S. cerevisiae* cells towards the pressure node, while the unaffected MS2 bacteriophage was directed towards a different outlet. The findings indicated that over 90% yields of MS2 were obtained while 80% of the *S. cerevisiae* were eliminated. Additionally, Fong et al. (2014) devised a novel channel structure for extracting cell-free dengue viruses (50 nm) from human lymphocytes (5–8 μm) using BAWs (Locatelli 2021). Another fluidic channel was constructed adjacent to the primary channel, forming a thin silicon wall referred to as a “transparent wall” to uncouple the acoustic and fluidic boundaries (Fong et al. 2014). This created asymmetrical pressure nodes in the fluidic channel, pushing cells further into the other half-channel, leading to improved separation. The outcome showed 98% separation purities for dengue viruses and 70% for human lymphocytes.

### Separation of proteins

Proteins exhibit a broad spectrum of biological functions as a class of macromolecules. Efficient protein sorting and aggregation are critical objectives in the realm of protein biotechnology (Chiti and Dobson 2006). In traditional acoustofluidic platforms, acoustic radiation force (ARF) is inadequate for manipulating proteins directly due to their small size. In contrast, Ding et al. (2013) utilized surface acoustic waves (SAWs) on supported lipid bilayers (SLBs) to rearrange proteins on planar SLBs effectively. Coupling SAWs with SLBs resulted in the modulation of membrane density, facilitating lipid transport and accumulation (Ding et al. 2013). The same setup could be employed for patterning proteins that were immobilized onto the SLB using binding methods such as biotin-avidin, electrostatic, and hydrophobic interactions. SAW pattern shifts were accomplished by tuning the two interdigital transducers with slightly different frequencies, leading to lipid and protein transport (Janshoff et al. 2000). Additionally, protein separation occurred when two different proteins were deposited onto the same SLB, possessing differing molecular weight, isoelectric point, and crystallization ability. Their competition for the antinode position caused the observed separation, even for similar-sized proteins such as streptavidin and avidin that differed in their crystallization ability. As an example, Ahmad et al. (2017) accomplished the separation of thrombin from mCardinal2 and human serum samples by utilizing aptamer-functionalized PS beads to trap the proteins in a TSAW device successfully. The authors could release the separated proteins from the microparticles for subsequent analysis by decreasing the solution’s temperature to below the solubility temperature of the polypeptides (Hassouneh et al. 2012). They demonstrated the efficacy of their technique by using streptavidin spiked in blood plasma as a model protein, achieving a separation efficiency over 90% with a detection limit of 0.75 nM and a release efficiency greater than 75% (Materón et al. 2021). While thrombin and IgE proteins were captured by two types of PS microparticles coated with apt15 and aptD17.4, respectively, mCardinal2 proteins remained unbound. The mixture was infused into the microchannel, and ARF separated the three proteins based on their size differential (Afzal et al. 2021).

### Separation of exosomes

Exosomes are small extracellular vesicles that cells secrete containing components of the parent cells, such as RNA, DNA, and protein, making them essential to intercellular communication (Meldolesi 2018, Xie et al. 2019a). Exosomes present in various body fluids are crucial for disease diagnosis and therapeutic purposes. Researchers have increased their focus on exosome separation technology from complex biofluids (Meldolesi 2018). Unlike traditional ultracentrifugation and filtration methods that encompass multiple operation steps, the acoustofluidic-based method enables continuous separation with less sample loss and lower potential for structural damage, offering a promising approach for exosome separation (Husseini et al. 2022). The small size of exosomes (40–160 nm) necessitates a high ARF for efficient separation in acoustofluidic and devised an SSAW nanofilter for effectively isolating exosomes (<200 nm) from larger extracellular microvesicles (MVs) present in cell culture media and stored RBC products101 (Lee et al. 2015). The cutoff size and separation performance were optimized by adjusting parameters such as channel design, acoustic transducer design, and flow rate. Wu et al. (2017) developed a multistage acoustofluidic device with two pairs of tilted-angle IDTs generating taSSAWs to isolate exosomes directly from whole blood102. The first module removed large blood components while the second exosome-isolation module achieved a 98.4% purity of exosomes from MV mixtures. The two modules working together achieved a 99.999% blood cell removal rate. The same device was used to study the impact of liquid viscosity on exosome separation103. It was also applied to successfully segregate exosomes from saliva samples.

**Fig. 5:**
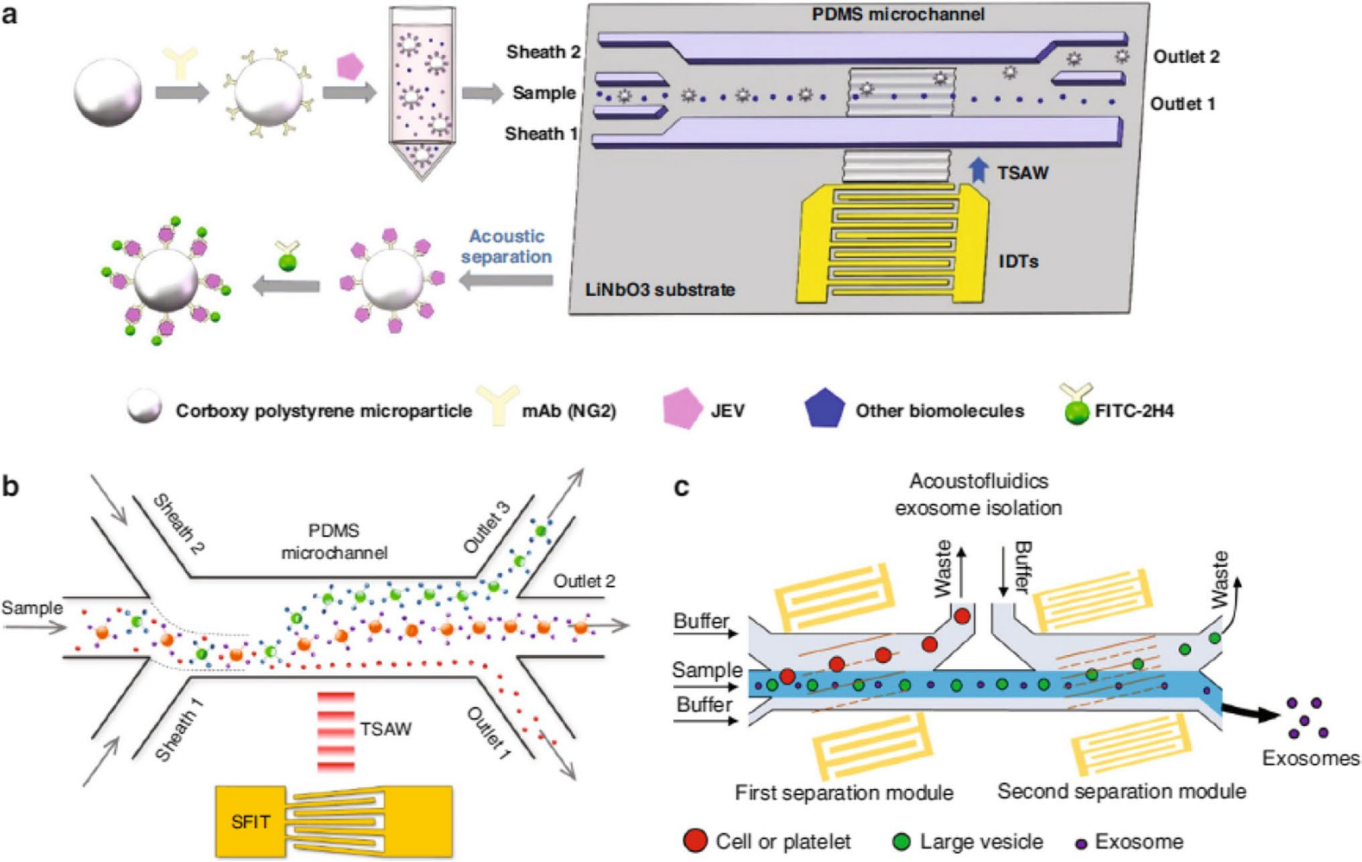
Acoustofluidic separation for bioparticles. **a** Isolation of Japanese encephalitis virus from intricate biological samples (Liu et al. 2020b). **b** Aptamer-coated microparticles and TSAW employed for triseparation of proteins from a mixed sample (Afzal et al. 2021). **c** A multistage acoustofluidic device used for separating exosomes from plasma samples (Wang et al. 2021)

Figure 5a is showing a schematic diagram in which demonstrates a method for isolating and detecting the Japanese encephalitis virus (JEV). Carboxy polystyrene microspheres, conjugated with an anti-JEV antibody, are used to capture the JEV in a virus solution. The solution is then pushed into a microchannel where interdigital transducers generate a high-frequency surface acoustic wave. This causes the PS-mAb-JEV composites to be deflected into another outlet. The separated composites are collected and labeled with FITC-conjugate anti-JEV antibody. Finally, the labeled composites are analyzed under a confocal microscope. This method allows for the isolation and detection of JEV using acoustofluidic driving and fluorescent labeling techniques.

Figure 5b illustrates an acoustofluidic method for triseparation of proteins conjugated with aptamer-coated microparticles in a microchannel. A slanted-finger interdigital transducer (SFIT) generates traveling surface acoustic waves (TSAWs) that exert an acoustic radiation force (ARF) on protein-loaded microparticles of different sizes. The acoustofluidic device consists of an SFIT deposited on a lithium niobate substrate and a polydimethylsiloxane (PDMS) microfluidic channel on top. The TSAWs propagate through the PDMS microchannel, interacting with the suspended protein-conjugated microparticles. The ARF varies depending on the size of the microparticles, causing them to flow along different streamlines and enabling triseparation of the proteins. The aptamers, attached to the microparticles via streptavidin-biotin linkage, were able to capture their target proteins (thrombin and immunoglobulin E). The resulting complexes, along with another protein (mCardinal2), were used to demonstrate the acoustofluidic triseparation of the proteins. This method achieved simultaneous separation of three different proteins (thrombin, immunoglobulin E, and mCardinal2) for the first time using TSAW-driven ARF in the acoustofluidic device (Afzal et al. 2021, Ahmed et al. 2018).

Figure 5c shows the concept of size-based isolation using an acoustofluidic device with two separation modules. The acoustic radiation force induced by the acoustic fields generated by the interdigital transducers (IDTs) around the channel is the driving force for this size-based isolation. The device includes two modules to remove particles larger than exosomes in a sequential manner. The IDTs in the first module generate a lower frequency acoustic field that is optimized for removing cell components and platelets. It is important to remove larger particles first as the higher frequency acoustic field of the second separation module has a greater influence on micrometer-sized particles, causing them to experience a larger acoustic radiation force. If larger particles are not removed first, they can block the channel and hinder the smooth operation of the device. Therefore, the first separation module is necessary to eliminate large particles before proceeding to the second module. The second module generates a higher frequency acoustic field designed to remove apoptotic bodies and microvesicles, which are larger than exosomes (Wang et al. 2021, Wu et al. 2021).

## Conclusions

In this article, an overview of the theories and mechanisms of acoustofluidic separation technology is presented. Most separation methods are relying on variations in particle sizes, but acoustofluidic separation is based on impedance and density which is feasible for particles of similar size. This technology has been widely demonstrated in biological fields, including separating different types of cells and bionanoparticles. While acoustofluidic devices have several advantages, such as being contactless, biocompatible, and having high sorting efficiency, there are still challenges to overcome in clinical practice, basic research, and commercialization. Acoustofluidic separation has great potential in in vitro diagnosis and point-of-care testing, but current devices have inadequate throughput for quick processing of large amounts of samples. Optimization of the microchannel structure and 3D acoustic field design offers potential solutions to this problem. There are still technical limitations in bionanoparticle sorting, but integrating other methods such as immunoaffinity may be feasible. Numerical simulation and machine learning can assist in experimental design. Although a few acoustofluidic technologies have been commercialized, the production cost is still a significant factor for companies. Universal systems that are compatible with different sorting chips may attract more business investment to develop commercialized products. Currently, acoustofluidic separation devices and platforms have high efficiency, but many still require the use of pretreated samples, which can complicate the process and negatively affect sample quality. It is essential to separate targets directly from raw samples like whole blood, which not only simplifies the process but also establishes a direct link to relevant diseases, thus advancing the clinical application of this technology. In vitro diagnosis (IVD) and point-of-care testing (POCT) are the primary clinical applications of acoustic separation techniques, but developing a complete solution that can handle sample preparation, target separation, and biomarker detection is still required. The all-acoustic platform holds immense promise for instance, CTCs can be isolated directly from a patient’s whole blood sample using ARF, and then, the CTCs are lysed using the strong acoustic energy of the subsequent sonication module to expose DNA. The acoustic bubble microstreaming effect can also improve the fluid pumping and sample-reagent mixing, facilitating the detection process. However, current acoustofluidic devices’ throughput is insufficient for rapid processing of a large number of samples, which is a fundamental requirement for clinical applications. Optimizing the microchannel structure to increase the flow rate and parallelizing multiple units can potentially solve this issue. However, excessive throughput may reduce separation efficiency, so it is vital to balance sorting performance and throughput. Current acoustofluidic devices mainly focus on 2D (vertical or horizontal) separation, but 3D separation has not been extensively explored. A possible solution is to design a 3D IDT array that can focus 3D acoustic fields on the microfluidic channel, which can improve the accuracy and flexibility of particle manipulation, especially for separating multiple targets in complex samples. With the development of acoustic metamaterials with unique acoustic parameters such as negative refractive index, integrating them into microfluidic devices can manipulate and control sound waves in novel ways, further improving the accuracy and spatial resolution of acoustic separation. Sorting submicron bioparticles remains challenging as the current method of removing larger particles is ineffective with smaller bioparticles. Hence, combining it with other techniques like the immunoaffinity method can facilitate specific sorting. New approaches to enhance the separation resolution of submicron bioparticles must be developed. Evaluating performance with repeated experiments can be burdensome and take significant time. Although simulation models like numerical simulations have partially solved this issue, there are still discrepancies between simulations and experiments. Recently, Talebjedi (2023) showed the potential of using machine learning methods like artificial neural network (ANN) prediction platform and multiobjective optimization to optimize acoustic separation, aiding experimental design. Presently, only a few acoustofluidic devices are commercially available. Translating laboratory technology into practical instruments is also challenging as operating acoustofluidic separation requires auxiliary tools like function generators, power amplifiers, and fluid control equipment. While developments in electronic integrated circuit technology have allowed for the high integration of these components, the technology’s relatively specific and narrow application scenarios and limited market size are other reasons why companies hesitate to invest in it.
